# Beyond genomic variation - comparison and functional annotation of three *Brassica rapa* genomes: a turnip, a rapid cycling and a Chinese cabbage

**DOI:** 10.1186/1471-2164-15-250

**Published:** 2014-03-31

**Authors:** Ke Lin, Ningwen Zhang, Edouard I Severing, Harm Nijveen, Feng Cheng, Richard GF Visser, Xiaowu Wang, Dick de Ridder, Guusje Bonnema

**Affiliations:** Laboratory of Plant Breeding, Wageningen University, Droevendaalsesteeg 1, 6708 PB Wageningen, the Netherlands; Laboratory of Bioinformatics, Wageningen University, Droevendaalsesteeg 1, 6708 PB Wageningen, the Netherlands; Institute of Vegetables and Flowers, Chinese Academy of Agricultural Sciences, Beijing, China

## Abstract

**Background:**

*Brassica rapa* is an economically important crop species. During its long breeding history, a large number of morphotypes have been generated, including leafy vegetables such as Chinese cabbage and pakchoi, turnip tuber crops and oil crops.

**Results:**

To investigate the genetic variation underlying this morphological variation, we re-sequenced, assembled and annotated the genomes of two *B. rapa* subspecies, turnip crops (turnip) and a rapid cycling. We then analysed the two resulting genomes together with the Chinese cabbage Chiifu reference genome to obtain an impression of the *B. rapa* pan-genome. The number of genes with protein-coding changes between the three genotypes was lower than that among different accessions of *Arabidopsis thaliana*, which can be explained by the smaller effective population size of *B. rapa* due to its domestication. Based on orthology to a number of non-brassica species, we estimated the date of divergence among the three *B. rapa* morphotypes at approximately 250,000 YA, far predating *Brassica* domestication (5,000-10,000 YA).

**Conclusions:**

By analysing genes unique to turnip we found evidence for copy number differences in peroxidases, pointing to a role for the phenylpropanoid biosynthesis pathway in the generation of morphological variation. The estimated date of divergence among three *B. rapa* morphotypes implies that prior to domestication there was already considerably divergence among *B. rapa* genotypes. Our study thus provides two new *B. rapa* reference genomes, delivers a set of computer tools to analyse the resulting pan-genome and uses these to shed light on genetic drivers behind the rich morphological variation found in *B. rapa.*

**Electronic supplementary material:**

The online version of this article (doi:10.1186/1471-2164-15-250) contains supplementary material, which is available to authorized users.

## Background

Plants in the Brassica genus display extreme morphological diversity, from cauliflower and broccoli through cabbages and Brussels sprouts to turnips and oil crops. Almost all organs are used for consumption: heads of cabbages and leaves on non-heading vegetable types, inflorescences of cauliflowers, tuberized stems/hypocotyls and/or roots of kohlrabi’s, turnips and swede and enlarged seeds and seedpods of oil types. One of the most important Brassica species, *Brassica rapa*, also shows this extreme morphological divergence, likely selected for by plant breeders all over the world, with heading and non-heading leafy crops, turnips and both annual and biannual oil crops.

Next to its economic value, *B. rapa* is also of particular interest in the study of genome evolution, because of its recent genome triplication after divergence from the common ancestor of Arabidopsis and Brassica [1]. The genome sequence of the mesopolyploid crop species *B. rapa* ssp*. pekinesis* Chiifu, a Chinese cabbage, was published in 2011 as the first *B. rapa* reference genome [2]. Interestingly, most retained paralogous genes in this genome still show higher similarity to each other than to their orthologs in *A. thaliana*. Comparative mapping studies identified a putative ancestral karyotype of the current Arabidopsis and Brassica genomes, with 24 conserved chromosomal blocks, as well as an on-going process of biased gene loss called gene fractionation in three subgenomes of *B. rapa*[1, 3]*.* These subgenomes have been reconstructed by grouping the 24 conserved blocks: the least fractionated subgenome (LF), with the highest gene densities; the medium fractionated subgenome (MF1), with moderate gene densities; and the most fractionated subgenome (MF2), with the lowest gene densities [3, 4].

**Figure 1:**
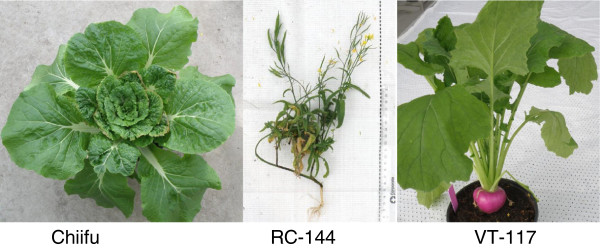
**Three** ***Brassica rapa*** **plants.** Left: the Chinese cabbage cultivar, Chiifu; middle: an oil-like rapid cycling line (RC-144); right: Japanese vegetable turnip (VT-117).

Our main research goal is to understand the genetic drivers underlying the enormous morphological variation between *B. rapa* subspecies. In this study, we therefore consider two *B. rapa* genomes – those of a vegetable turnip double haploid line (DH-VT117) and a rapid cycling inbred line (RC-144) – as representatives of the very distinct morphotypes turnip and annual oil (Figure 1). The vegetable turnip has an enlarged hypocotyl/root, whereas the rapid cycling line is developed in Wisconsin by intercrossing mainly annual oils and pakchois/caixins and selecting for earliness in flowering [5]. As recent studies suggested that the genome-wide density of variants is much higher between accessions of one plant species than between lines in one mammalian species, in this study we not only resequenced the turnip and rapid cycling line genomes, but also assembled and re-annotated them, resulting in two new reference genomes [6–11].

**Figure 2:**
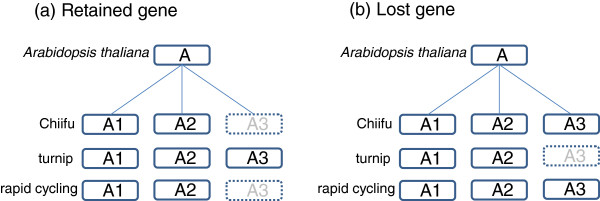
**Definition of retained and lost genes.** Illustrative examples of a retained and a lost gene in turnip. **(a)** *A. thaliana* gene A has three orthologous genes in turnip, but only two in Chiifu and rapid cycling; hence, we call A a retained gene for turnip based on the presence of A3. **(b)** Gene A is considered a lost gene for turnip based on the absence of A3.

These two new genomes were combined with the reference Chiifu genome (representative of the heading leafy type) to form an initial *B. rapa* pan-genome. This concept was raised first in the study of bacterial species, to define the full complement of genes of several closely related strains [12]. In a pan-genome, we can distinguish *common* genes, present in all accessions of a species; *dispensable* genes, occurring in more than one genome; and *unique* genes, specific to a single genome [13]. In the *B. rapa* pan-genome, we find such genes and explore functional annotations of the unique gene set to find morphotype-specific genes. We also analyze the orthology of genes in the pan-genome to *Arabidopsis thaliana* and *Thellungiella halophila* to find lost genes (orthologs missing in one of the three *B. rapa* genomes) and retained genes (orthologs present in only one of the genomes) (Figure 2). Finally, using orthologous genes we estimate the divergence date of the three *B. rapa* species and find that it far precedes domestication. The two newly assembled and annotated genomes are available to the community as an online resource at http://www.bioinformatics.nl/brassica/turnip and http://www.bioinformatics.nl/brassica/rapid-cycling, accompanied by the tools developed to explore the pan-genome.

## Results

### MAKER re-annotation of the reference genome Chiifu

**Figure 3:**
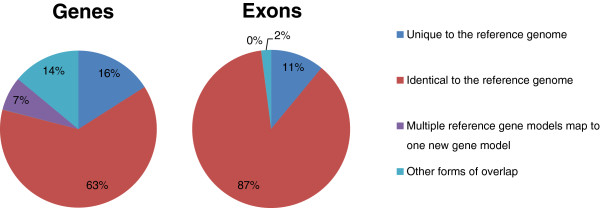
**Coverage of published Chiifu reference gene models compared with re-annotated Chiifu gene models.** Coverage of published Chiifu reference gene models based on number of genes and exons compared with those re-annotated by MAKER, considering a prediction identical when overlapping the reference gene model by at least 75%.

To get a comparable genome annotation for all three *B. rapa* species, we first re-annotated the Chiifu reference genome using MAKER [14]. This re-annotation covered about 85% of the original 41,019 gene models found in the Brassica database (version 1.2), resulting in 41,052 gene models of which 11,715 were novel predictions (Table 1) [15]. The re-annotation covered about 90% of the exons from the Brassica database (Figure 3). The remaining 6,437 exons, roughly 10%, were mainly (5,615) located in low complexity regions of the genome. As expected, when we decreased the minimum overlap required for matching gene models, the number of recovered gene models increased: only five genes with short lengths (<200 bp) were still missing if the minimum overlap required was 10%. Approximately 75% of re-annotated gene models could be assigned a Gene Ontology term [16].

**Table 1:** **Comparisons of Chiifu gene models made by MAKER and obtained from BRAD**

		BRAD_only	MAKER_only	Overlap_reciprocal	Overlap_split	Overlap_join	Overlap_total
100%	Gene	8,500	26,118	11,271	156	2,880	32,519
	Exons	30,121	42,416	164,479	26	895	176,454
75%	Gene	6,437	11,715	25,848	187	2,815	34,582
	Exons	21,838	34,590	179,737	47	964	184,743
50%	Gene	5,229	8,601	29,857	203	2,762	35,790
	Exons	19,385	31,639	184,909	78	1,007	187,198
25%	Gene	4,239	6,544	33,617	719	2,719	36,780
	Exons	18,270	30,178	187,577	347	1,039	188,313

Four different minimum overlap requirements (expressed as a fraction of the Chiifu reference gene model) used to compare two gene models at both gene level and exon level. The *BRAD_only* and *MAKER_only* columns represent features found only in the reference gene model and MAKER generated gene model respectively. Intersections between two gene models mainly include the fraction overlap reciprocal for both (*overlap_reciprocal*), overlaps that split one reference feature to many MAKER features (*overlap_split*) or join many reference features to one MAKER feature (*overlap_join*).

### Genomic variation between the Chinese cabbage, turnip and rapid cycling genotypes

**Figure 4:**
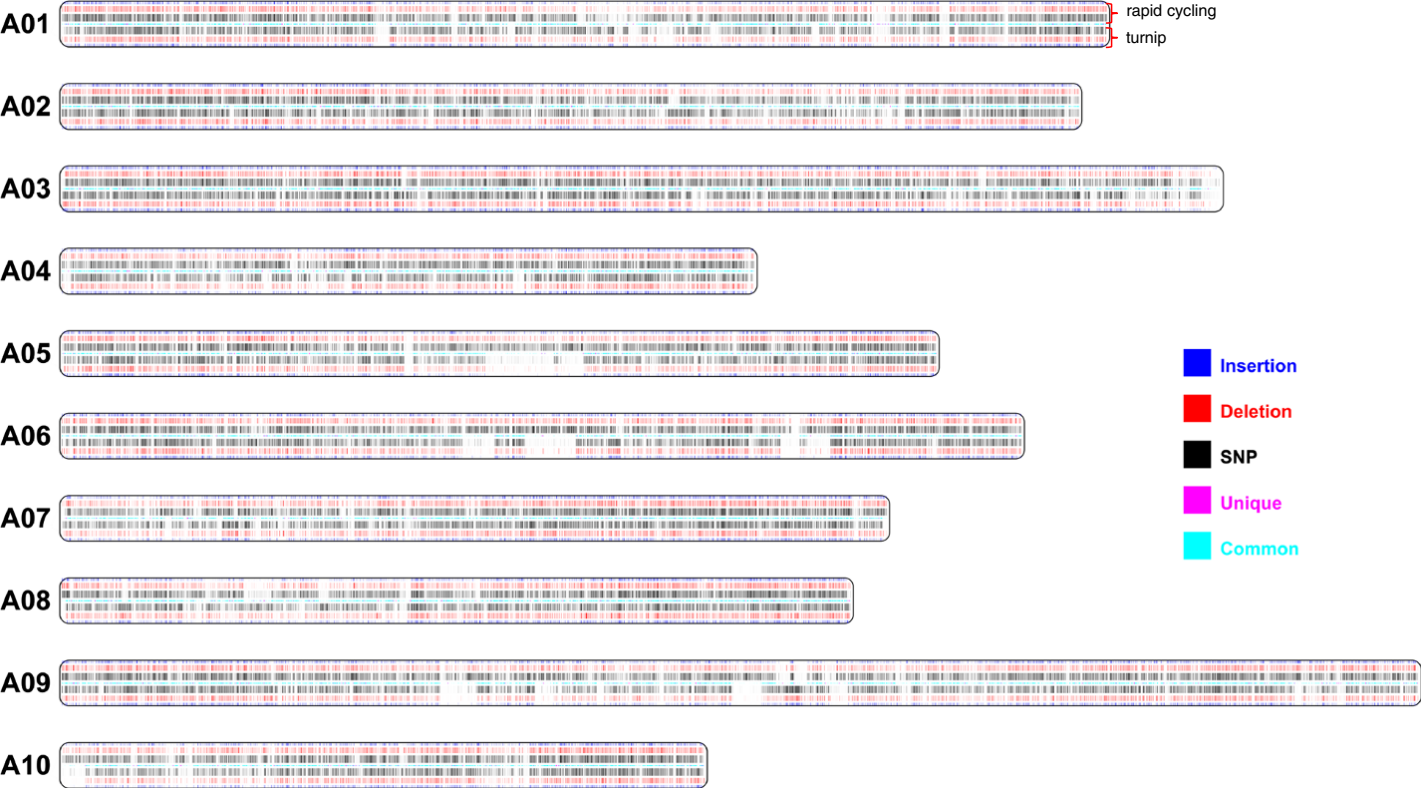
**Genomic variations anchored to chromosomes in resequenced turnip and rapid cycling genomes.** Genomic variants including insertions, deletions and SNPs between resequenced turnip, rapid cycling and reference Chiifu genome on each chromosome. On each chromosome (A01-A10), the middle row represents either common or unique variations in the Chiifue genome. Genomic variations between rapid cycling and Chiifu are presented in the top three rows, variations between turnip and Chiifu in the bottom three rows. Common variations have the same sequence composition at the same position in both rapid cycling and turnip; unique variations have different nucleotides between the three genomes at the same position.

Within a species, the genomic variation between subspecies can vary substantially, as shown in several published intra-species comparative genomic studies [6, 8, 10]. We mapped the turnip and rapid cycling resequenced genomes to the reference Chiifu genome and identified 1,137,171 and 1,308,697 genomic variants respectively (Figure 4). This is less variation than between pairs of *A. thaliana* accessions [12]. There are 596,323 genomic variations common relative to the reference Chiifu genome (turnip and rapid cycling share the same allele at those sites), 539,747 genomic variations only found between turnip and Chiifu and 711,273 genomic variations only found between rapid cycling and Chiifu. Only 1,101 genomic variations (458,377 bps) are unique, i.e. differ between all three (re) sequenced genomes.

### Re-annotation of turnip and rapid cycling

**Figure 5:**
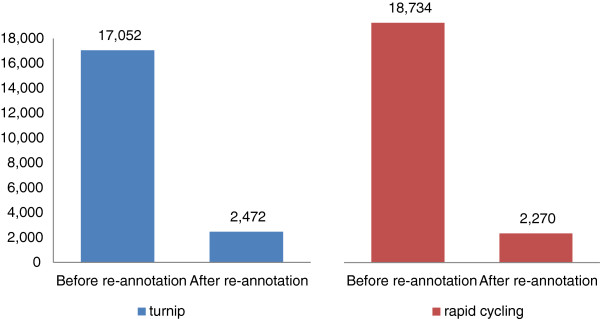
**Number of genes predicted to be functionally affected by genomic variants.** Before annotation, genes were considered functionally affected in the rapid cycling line or in turnip when one of the following variants was found w.r.t. the Chiifu genome: SPLICE_SITE_ACCEPTOR, SPLICE_SITE_DONOR, START_LOST, EXON_DELETED, FRAME_SHIFT, STOP_GAINED or STOP_LOST. Genes were considered affected if they had no orthologous gene at the same chromosome/scaffold of Chiifu genome after its re-annotation.

The genome sequences of turnip and rapid cycling were reconstructed by applying all genomic variation found to the reference genome. The total lengths of both resulting genomes were almost the same as that of the reference genome (283.84 Mbs): 282.93 Mbs for turnip and 282.69 Mbs for rapid cycling. Before re-annotation of the reference genomes, nearly half of its gene models appeared to be affected by changes in the protein coding region in either turnip (17,052) or rapid cycling (18,734) with moderate to high impact [17] (Additional file 1). Re-annotation of the turnip and rapid cycling genomes resulted in 40,708 and 40,506 predicted gene models respectively, slightly below the number found in the reference genome (41,052). After re-annotation, the number of genes found in the turnip and rapid cycling genomes which changed function or became pseudogenes with respect to the Chiifu genome was only 2,472 resp. 2,270 (Figure 5).

### Pan-genome construction and detection of retained and lost genes

**Figure 6:**
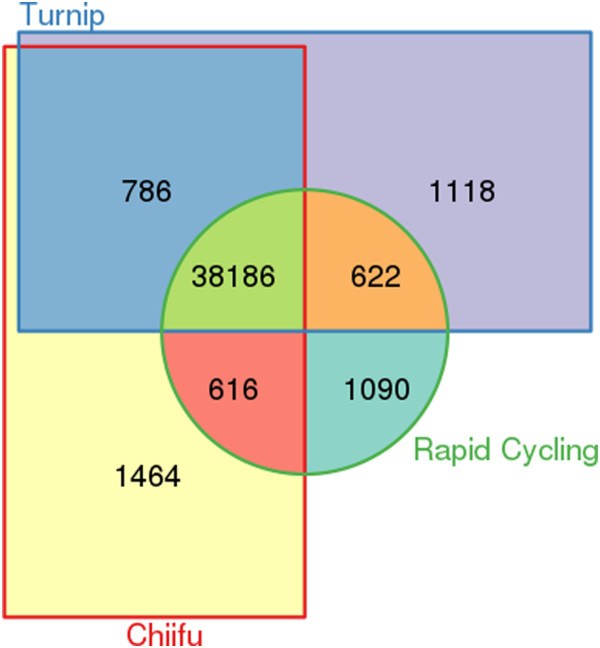
***B. rapa*** **pan-genome composition.** There are 38,186 genes classified as common in the B. rapa pan genome; the number of unique genes was 1,464 in Chiifu, 1,118 in turnip and 1,090 in rapid cycling.

Most of the genomic variation maps to intergenic regions, followed by introns, exons and UTRs (Table 2). After re-annotation of the rapid cycling and turnip genomes, 38,186 genes are found to be common, i.e. present in all three genomes (the pan-genome) while 1,090, 1,118 and 1,464 genes are unique to turnip, rapid cycling and Chiifu respectively (Figure 6). Functional annotation of these genes resulted in 172,430 Gene Ontology (GO) assignments to 30,976 genes (Additional file 2).

**Table 2:** **Number of genomic variants located in exons, introns, UTRs and intergenic regions over three subgenomes**

	Turnip			Rapid cycling		
Type (alphabetical order)	LF	MF1	MF2	LF	MF1	MF2
EXON-Count	80,093	36,695	44,991	89,042	42,165	49,868
EXON-Length	170,181	138,159	101,503	238,506	112,953	122,966
INTERGENIC-Count	243,148	129,852	179,159	280,600	154,959	192,416
INTERGENIC-Length	620,502	355,733	460,022	745,887	447,983	534,174
INTRON-Count	138,428	69,785	83,492	160,185	80,189	94,772
INTRON-Length	312,855	160,185	179,874	357,133	166,618	216,723
UTR-Count	8,837	4,816	5,995	10,340	5,673	6,619
UTR-Length	20,918	9,232	11,538	34,871	11,044	12,382

Genomic variants mapped on four different types of genome regions grouped by three subgenomes (LF, MF1, MF2) in turnip and rapid cycling. The counts indicate the number of variations in each genomic region; the length is the sum over all genomic variations. SNPs are defined as being 1 bp long.

**Figure 7:**
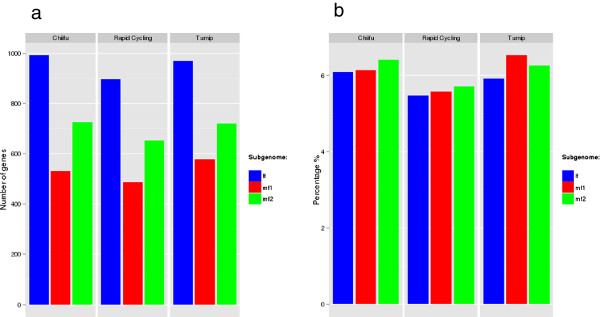
**Subgenome composition of dispensable and unique genes in three** ***B. rapa*** **genotypes.** The subgenome composition of dispensable and unique genes in three *B. rapa* genotypes in terms of **(a)** number of genes; **(b)** frequency of gene changes, calculated as number of changed genes divided by the total number of total genes in the subgenome. LF: less fractionated subgenome, with the highest gene densities; MF1: more fractionated subgenome 1, with moderate gene densities; MF2: most fractionated subgenome 2, with lowest gene densities.

In total, we thus found 3,672 unique genes, found in only one of the three genomes; all remaining non-unique, non-common genes we called dispensable. About 1,443 out of 2,526 unique and dispensable genes in turnip could be annotated with at least one GO term, as was the case for 1,366 out of 2,328 genes in rapid cycling and 1,649 out of 2,866 in Chiifu. Most of these genes were assigned to only ten biological process GO terms, seven of which were common to the three genomes (Table 3 and Additional file 3). Gene models predicted from contigs that could not be mapped against the Chiifu genome were annotated separately. The number of genes thus found with at least one GO term annotation was 918 for the turnip genome and 548 for the rapid cycling genome (Additional file 4). Most unique and dispensable genes mapped to the LF subgenome, the least mapped to the MF1 subgenome. Corrected for total gene count, the proportion of genes affected by changes to their protein coding region is lowest in the LF subgenome (Figure 7).

**Table 3:** **Top ten GO biological processes with most genes assigned in Chiifu, turnip and rapid cycling**

Chiifu		Turnip		Rapid cycling	
Biological process	#genes	Biological process	#genes	Biological process	#genes
Response to stress	40	Response to stress	65	Response to stress	31
Response to abiotic stimulus	31	Protein modification process	35	Protein modification process	16
Response to endogenous stimulus	18	Catabolic process	32	Response to abiotic stimulus	15
Secondary metabolic process	16	Transport	30	Signal transduction	13
Signal transduction	16	Response to abiotic stimulus	29	Cellular component organization	13
Catabolic process	14	Signal transduction	27	Response to biotic stimulus	12
Cellular component organization	13	Response to biotic stimulus	21	Transport	12
Anatomical structure morphogenesis	11	Carbohydrate metabolic process	18	Catabolic process	11
Response to biotic stimulus	11	Cellular component organization	17	Response to endogenous stimulus	9
Protein modification process	10	Response to endogenous stimulus	14	Lipid metabolic process	9

Only dispensable and unique genes were included in the analysis. The term “response to stress” is the most over-represented and seven out of these ten GO terms are found in Chiifu, turnip as well as rapid cycling.

To study gene copy number variation in the *B. rapa* pan-genome, orthology between genes in *B. rapa* and its close relatives *Arabidopsis thaliana* and *Thellungiella halophila* was computed. For the common gene set we found 19,301 orthologs in *A. thaliana* and 20,825 in *T. halophile*. The numbers of genes with one, two or three orthologous genes in *B. rapa* were 15,200/3,650/690 in *A. thaliana* and 17,800/2,950/520 in *T. halophila* (Table 4). Further analysis of the *B. rapa* dispensable and unique genes with orthologous genes in either *A. thaliana* or *T. halophila* showed that the rapid cycling line and turnip DH line had over 40% resp. 30% less retained genes w.r.t. *A. thaliana* than Chiifu, yet an approximately equal number of lost genes (Table 5).

**Table 4:** **Orthologous genes of** ***Arabidopsis thaliana*** **and** ***Thellungiella halophila*** **found in Chiifu, turnip and rapid cycling**

Number of copies in ***B. rapa***	1		2		3	
Compared genomes	at	th	at	th	at	th
Chiifu	15,237	17,880	3,706	3,041	691	540
Turnip	15,190	17,812	3,676	2,907	713	503
Rapid cycling	15,225	17,774	3,595	2,960	681	519

The number of *A. thaliana* (“at”) and *T. halophile* (“th”) genes having one, two and three copies of orthologous genes in Chiifu, turnip and rapid cycling.

**Table 5:** **Retained and lost genes in Chiifu, turnip and rapid cycling**

	Chiifu	Turnip	Rapid cycling
Genes with copy number changes	1,151	1,053	932
Retained genes	265	180	156
Lost genes	886	873	906
Retained|lost gene assigned to gene families	40|23	35|19	33|20
Genes present in unique and dispensable gene set, without at or th orthologs*	231	280	336

Dispensable and unique genes having orthologs in *A. thaliana* or *T. halophila* were included to determine the retained and lost genes. The latest curated gene family assignment of *A. thaliana* genes from TAIR was used.

* The number of *B. rapa* unique and dispensable genes without *A. thaliana* (“at”) or *T. halophile* (“th”) orthologous genes.

### Functional annotation of dispensable and unique genes in the Chiifu, turnip and rapid cycling genomes

The unique and dispensable genes can be placed in 87 KEGG pathways in turnip, 104 in rapid cycling and 89 in Chiifu, with starch and sucrose metabolism containing the largest proportion of genes (19 in turnip, 20 in rapid cycling and 13 in Chiifu) [18]. These genes are also found in 97, 102 resp. 94 plant-specific pathways hosted by the Plant Metabolic Network in PlantCyc [19]. Genes are much more scattered over different metabolic pathways in PlantCyc than in KEGG [18, 19]. The number of genes found in any pathway in PlantCyc (119 in turnip, 123 in rapid cycling and 112 in Chiifu) is less than half of the number of genes found in KEGG (285 in turnip, 297 in rapid cycling and 254 in Chiifu) since fewer enzymes are associated with each PlantCyc metabolic pathway (Additional file 5).

GO enrichment analysis shows that the dispensable and unique genes in Chiifu have both the most overrepresented (59) and underrepresented (50) GO terms, while the dispensable and unique genes in rapid cycling have the least overrepresented (11) and underrepresented (16) terms and turnip has 35 overrepresented and 13 underrepresented terms. The number of genes assigned to enriched GO terms is higher in turnip (1,095) than in Chiifu (823) and rapid cycling (704).

### Genes with association to different morphotypes

**Figure 8:**
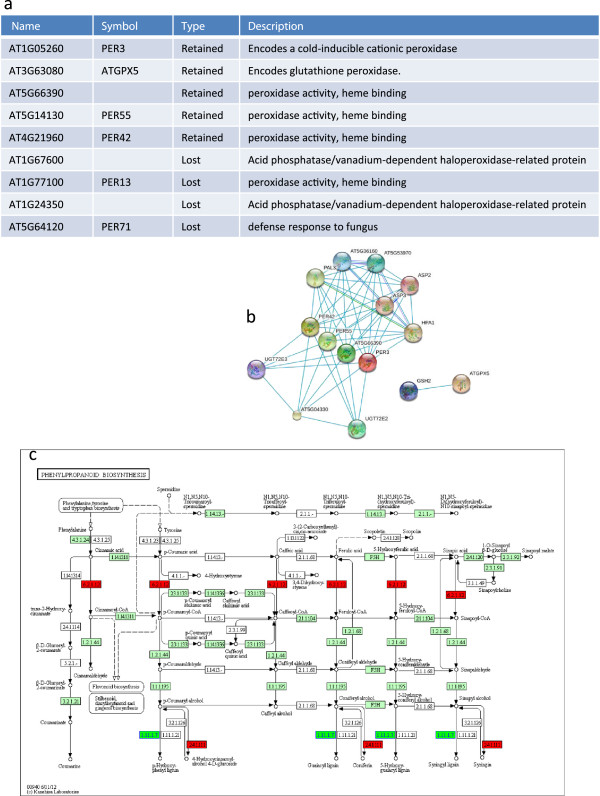
**Network analysis of retained and lost genes in turnip.** 155 *A. thaliana* peroxidase-related genes were selected. **a)** Five retained genes and four lost genes were identified in turnip, five of which were class III peroxidases. **b)** Summary of the functional protein interaction network found by STRING using five retained genes as input. **c)** Phenylpropanoid biosynthesis pathway in *A. thaliana,* including four retained genes and two lost genes. *A. thaliana* genes that encode enzymes are indicated by light green colored boxes; red resp. dark green boxes indicate genes with less resp. more copies in rapid cycling than in Chiifu and turnip.

Next, we specifically looked for genes potentially related to morphological variation, by considering retained and lost genes with orthologs in both *A. thaliana* and *T. halophila*. Only a small percentage of these, 15% of the retained and 10% of the lost genes, could be categorized into known *A. thaliana* gene families (Table 6). The set of unique and dispensable genes found in turnip is enriched for the GO cellular component term “peroxisome”, and contains Class III peroxidases among both lost (AT5G64120) and retained (AT1G05260 with gene symbol RCI3) genes. To refine our understanding of a possible role of peroxidases in turnip formation, we more closely investigated *B. rapa* genes orthologous to 155 peroxidase related genes in *A. thaliana*[20]. We exploited synteny information to support the confidence in orthology predictions and to help distinguishing true orthologs, since *A. thaliana* and *B. rapa* are evolutionary very close [21]. *B. rapa* orthologs of five *A. thaliana* genes were retained and of four *A. thaliana* genes were lost in turnip compared to Chiifu and rapid cycling (Figure 8a). We found proteins functionally interacting with these genes using STRING (Figure 8b) [22]. Four of the five retained genes were involved in the phenylpropanoid biosynthesis pathway and the fifth, AT3G63080 (ATGPX5), a glutathione peroxidase, may contribute to glutathione synthesis. Only one of the four *A. thaliana* orthologs of the lost genes, AT5G64120 (PER71), was predicted to interact with other proteins in STRING, whereas both PER71 and another lost gene AT1G77100 (PER13) are also involved in phenylpropanoid biosynthesis. We then examined all genes known to be involved in the phenylpropanoid biosynthesis pathway in *A. thaliana* and found that while orthologs of genes encoding a peroxidase (EC number 1.11.1.7) were enriched in turnip, genes encoding a 4-coumarate-CoA ligase (EC 6.2.1.12) or a coniferyl-alcohol glucosyltransferase (EC 2.4.1.111) were underrepresented. The six *A. thaliana* genes encoding this ligase have ten orthologs in the common gene set of *B. rapa*, but only two *B. rapa* genes are orthologous to three *A. thaliana* genes coding for the glucosyltransferase. This suggests the lower copy number of genes in turnip coding for the glucosyltransferase may cause the reduction of 4-D-glucoside, coniferin, syringin and hence increase the production of different lignins (Figure 8c).

**Table 6:** **Gene family assignment for retained and lost genes in Chiifu, turnip and rapid cycling**

Chiifu				Turnip				Rapid cycling			
TAIR gene family description	Class	Number of genes in common sets	Number of genes in unique and dispensable sets	TAIR gene family description	Class	Number of genes in common sets	Number of genes in unique and dispensable sets	TAIR gene family description	Class	Number of genes in common sets	Number of genes in unique and dispensable sets
C2H2 transcription factor family	LOST			Acyl lipid metabolism family	LOST			C3H transcription factor family	LOST		
Core DNA replication machinery	LOST			C2H2 Transcription factor family	LOST			Cytochrome P450	LOST		
Cytochrome P450	LOST			Class III peroxidase	LOST			Cytoplasmic ribosomal protein gene family	LOST		
Cytoplasmic ribosomal protein gene family	LOST			Cytochrome P450	LOST			Glutathione S-transferase family	LOST		
Cytoskeleton	LOST			Cytoplasmic ribosomal protein gene family	LOST			Glycoside hydrolase gene families	LOST		
EF-hand containing proteins	LOST			EF-hand containing proteins	LOST			Glycosyltransferase gene families	LOST		
Expansins	LOST			FH2 proteins	LOST			Homeobox transcription factor family	LOST		
Glutathione S-transferase family	LOST			Glycosyltransferase gene families	LOST			Inorganic solute cotransporters	LOST		
Glycosyltransferase gene families	LOST			MIP family	LOST			Lipid metabolism gene families	LOST		
Lateral organ boundaries gene family	LOST			Miscellaneous membrane protein families	LOST			MAP kinase kinase kinase kinase (MAPKKKK) family	LOST		
Miscellaneous membrane protein families	LOST			Monosaccharide transporter-like gene family	LOST			Miscellaneous membrane protein families	LOST		
MYB Transcription factor family	LOST			Trihelix transcription factor family	LOST			Primary pumps (ATPases) gene families	LOST		
Primary pumps (ATPases) gene family	LOST			ARF transcription factor family	RETAINED	15	1	Receptor kinase-like protein family	LOST		
Acyl Lipid metabolism family	RETAINED	587	3	Carbohydrate esterase gene families	RETAINED	75	1	Acyl Lipid metabolism family	RETAINED	587	4
AP2-EREBP transcription factor family	RETAINED	170	1	Chloroplast and mitochondria gene families	RETAINED	53	1	BZR transcription factor family	RETAINED	6	1
ARF transcription factor family	RETAINED	15	1	Class III peroxidase	RETAINED	71	1	CBL-interacting serione-threonine Protein Kinases	RETAINED	24	1
C2H2 transcription factor family	RETAINED	215	1	Cytochrome P450	RETAINED	144	2	Core cell cycle genes	RETAINED	65	1
C3H transcription factor family	RETAINED	162	1	Cytoplasmic ribosomal protein gene family	RETAINED	221	1	Cytochrome P450	RETAINED	144	3
CCAAT-HAP3 transcription factor family	RETAINED	13	1	GeBP transcription factor family	RETAINED	10	1	Glycoside hydrolase gene families	RETAINED	335	1
Cytoplasmic ribosomal protein gene family	RETAINED	221	2	Glutathione S-transferase family	RETAINED	37	2	Glycosyltransferase gene families	RETAINED	280	1
EF-hand containing proteins	RETAINED	175	2	Glycosyltransferase gene families	RETAINED	280	1	Histidine phosphotransfer proteins	RETAINED	5	1
Eukaryotic initiation factor gene family	RETAINED	101	1	HSP70s	RETAINED	4	1	Inorganic solute cotransporters	RETAINED	93	1
Glutathione S-transferase family	RETAINED	37	1	Lipid metabolism gene families	RETAINED	106	2	Lipid metabolism gene families	RETAINED	106	1
Glycoside hydrolase gene families	RETAINED	335	2	MADS-box transcription factor family	RETAINED	89	2	Miscellaneous membrane protein families	RETAINED	415	3
Glycosyltransferase gene families	RETAINED	280	1	Myosin	RETAINED	17	1	NAC transcription factor family	RETAINED	97	1
Lateral organ boundaries gene family	RETAINED	37	2	Organic solute cotransporters	RETAINED	272	1	Nodulin-like protein family	RETAINED	65	3
MAP kinase kinase kinase family	RETAINED	74	1	Receptor kinase-like protein family	RETAINED	237	4	Organic solute cotransporters	RETAINED	272	2
MIP family	RETAINED	37	1	REM transcription factor family	RETAINED	20	1	Pollen coat proteome	RETAINED	3	1
Miscellaneous membrane protein families	RETAINED	415	2					RCI2 gene family	RETAINED	6	2
MYB transcription factor family	RETAINED	154	1					SNAREs	RETAINED	66	1
Plant defensins superfamily	RETAINED	6	1								
Plant U-box protein (PUB)	RETAINED	66	1								
Primary pumps (ATPases) gene family	RETAINED	31	1								
Receptor kinase-like protein family	RETAINED	237	2								

Overview of lost and retained genes assigned to *A. thaliana* gene families. The “LOST” gene family has no value in the dispensable and unique gene set because the inconsistency of counts in the other two genotypes.

### Estimation of divergence date between turnip, rapid cycling and Chiifu

We found 7,768 orthologous gene sets in *A. thaliana*, *Arabidopsis lyrata*, *Oryza sativa*, *Vitis vinifera* and *Zea mays* using the latest OMA browser [23] dataset (March 2012); 1,714 of these remained after filtering on a 1:1 orthologous relationship. Combining this set of 1,714 remaining genes with orthologous groups in the *B. rapa* pan-genome left 104 groups of orthologous genes with a meaningful OMA group description. These were used to infer the divergence date among the three *B. rapa* genomes, at around 0.259 MYA.

### Availability

**Figure 9:**
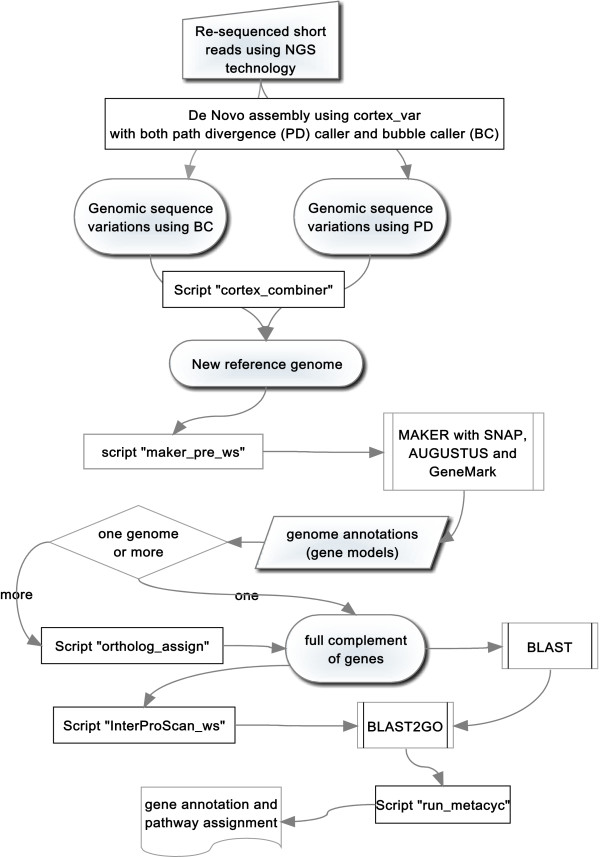
**Workflow of the study.** The workflow describes the methods and logic used in the study, from raw sequence reads to the annotation of the full complement of genes in a genome. Newly created scripts are marked by “Script”. Any number of genomes can be analyzed using this workflow, provided there is sufficient computational power.

The two newly-assembled genomes representing the turnip morphotype (turnip) and the oil crop morphotype (rapid cycling), their annotation files, a gene list for the three categories of pan genomes and the Blast2GO project files generated in the study are all provided (Additional files 6, 7, 8 and 9). The genomes can also be browsed at http://www.bioinformatics.nl/brassica/turnip and http://www.bioinformatics.nl/brassica/rapid-cycling. All used software tools in this project can be handled by biologists with some basic bioinformatics skills and the pre-/post-processing scripts are available for download (Table 7, Figure 9 and Additional file 10). These programs were run on an OpenSuSE Linux server with 16 AMD Opteron Processor cores and 128 GB of memory.

**Table 7:** **Software and scripts used in the project**

Name	Running time (h)	Input format	Output format	Script purpose
cortex_var	24 / genotype	Fastq	vcf	-
*cortex_combiner	< 1	Vcf	fasta	Post-processing of cortex
*maker_pre_ws	24	Txt	Fasta	Pre-processing for MAKER
MAKER	140/genotype	Fasta	gff, fasta	-
*ortholog_assign	20/genotype	Fasta	csv, fasta	Post-processing of MAKER
NCBI BLAST	200	Fasta	xml	-
*InterProScan_ws	100	Fasta	xml	Pre-processing for Blast2GO
Blast2GO	< 1	Xml	csv	-
*run_metacyc	< 0.1	Txt	csv	Post-processing of Blast2GO
*beast_pre	< 5	Txt	nex	Pre-processing of BEAST
BEAST	24	Nex	png	-
*choose_fasta	< 0.1	Txt	Fasta	Extract sequence from fasta

The order in the table indicates the flow of the analysis, except for the script “choose_fasta” which can be used anytime when needed. Names starting with an asterisk are scripts generated specifically for this work. The script purpose column indicates when the scripts should be used before or after certain program. All scripts run under Linux and provide a short usage summary when started without arguments. “txt” input format: a list of file names used for the scripts.

## Discussion

### Variability in the B. rapa pan-genome

The three *B. rapa* genomes considered in this work – Chiifu, turnip and rapid cycling – differ by about 0.45 per 100 base pairs, considerably less than the differences between lines of maize (1-2/100 bp) but very close to differences between various accessions of *A. thaliana* (0.5/100 bp) and of rice (0.4/100 bp) [11, 24, 25]. To further investigate the pan-genomic variation, we focused on the unique genes, on average 1,224 per *B. rapa* genome. The frequency of functional unique genes over the three subgenomes agrees with the theory that one of the subgenomes (LF) is dominant and hence has the lowest percentage of affected genes (Figure 7).

We expected the number of unique genes in each *B. rapa* genome to be larger than the average number of unique genes found in different *A. thaliana* accessions, mainly because the morphological variation between a tuber forming turnip, a heading cabbage and an oilseed rapid cycling is larger than between, say, three *A. thaliana* accessions. Additionally, the recent genome triplication in *B. rapa* may have lowered selective pressure on a subset of the genes. However, a recent study analyzing 18 *A. thaliana* accessions found only 319 unique genes per accession on average. Such a comparison is not completely fair however, as the *A. thaliana* comparisons used a different definition of unique gene. We thus selected the three *A. thaliana* accessions (Can, Wil and Sf) with the highest number of genes with predicted major disruptions, and used the protein sequences of their gene models to find unique genes by exactly the same process as in our study. This yielded on average 1,700 unique genes, *higher* than the 1,224 found in the *B. rapa* genomes. One explanation may be that the effective population size is much higher for *A. thaliana* than for *B. rapa*, which went through several domestication bottlenecks. Additionally, the three *B. rapa* genotypes are all landraces (or intercrossed genotypes) growing in protected agricultural settings, with varieties selected by breeders and farmers, while *A. thaliana* is a weed that grows in natural environments under diverse abiotic and biotic stresses (drought, cold, pathogens) with different selection forces.

Our findings are also in line with a previous study, in which the genetic variation in a *B. rapa* core collection representing all morphotypes and geographical origins was analyzed based on molecular marker profiles [26, 27]. Bayesian clustering implemented in the STRUCTURE software revealed four subpopulations, each representing different morphotypes (I turnip accessions from European origin; II Asian leafy types like Pakchoi plus Asian turnips; III annual oil accessions and IV mainly accessions of Chinese cabbage (CC). AMOVA results indicated that the percentage of variation found within sub-populations/morphotypes is much larger (85%) than the variation among populations (15%), suggesting that only a small percentage of the polymorphisms relate to the sepcific observed morphological differences.

### Genomic determinants of morphological variation

Studying the functions of unique and dispensable genes could reveal whether they play a role in the extreme morphological differences between the three plants. Through functional annotation, we found that peroxidases are good candidates for genes involved in the definition of plant morphology. Peroxidases play a role in protection from biotic and abiotic stresses, but also in lignin formation. Four of five turnip specific retained *B. rapa* genes orthologous to *A. thaliana* peroxidases are involved in the phenylpropanoid biosynthesis pathway. Phenylpropanoids are a group of plant secondary metabolites and specific compounds differentially accumulate in particular tissues with specialized functions. These results suggest that lignin may be important for turnip tuber formation, which can relate to the increased numbers of xylem vessels in the turnip tuber.

In this paper we focus on the DNA level, but it is entirely possible that turnip formation is (additionally) regulated at the transcriptional or even post-translational level. Gene loss occurred more in rapid cycling than in turnip and Chiifu (906 in rapid cycling, 873 in turnip and 886 in Chiifu). Rapid cycling may have a different composition of flowering time genes because it was generated by crossing early flowering *B. rapa* genotypes to create a morphotype with a short life cycle for educational purposes [5]. To verify this hypothesis, we looked for genes in the three *B. rapa* genomes orthologous to 367 known flowering related *A. thaliana* genes. These flowering genes were classified into five different categories, including flower development, gibberellin-, photoperiod/circadian rhythm- and vernalization pathway and metabolic processes (Additional file 11). In rapid cycling, there are five lost genes related to flowering time (covering all five categories), compared to only three in Chiifu and turnip (from a single category, photoperiod in turnip and vernalization in Chiifu) (Table 8).

**Table 8:** **Flowering time related lost genes in three** ***B. rapa*** **genotypes**

Genotype	Ara ID	Gene name	Pathway	Gene full name	Protein function
Rapid cycling	AT2G32950	COP1	Photoperiod	CONSTITUTIVE PHOTOMORPHOGENIC 1	E3 ubiquitin ligase
Rapid cycling	AT3G11440	MYB65	Gibberellin	MYB65	MYB transcription factor
Rapid cycling	AT3G20740	FIS3	Vernalization	FERTILIZATION-INDEPENDENT ENDOSPERM	Encodes a protein similar to the transcriptional regular of the animal Polycomb group
Rapid cycling	AT5G03790	LMI1	Flower development	LATE MERISTEM IDENTITY 1	HD-Zip transcription factor
Rapid cycling	AT5G47010	LBA1	Metabolic process	LOW-LEVEL BETA-AMYLASE 1	Required for nonsense-mediated mRNA decay
Chiifu	AT1G04440	CKL13	Vernalization	CASEIN KINASE LIKE 13	Protein serine/threonine kinase activity
Chiifu	AT4G25470	CBF2	Vernalization	C-REPEAT/DRE BINDING FACTOR 2	Encodes a member of the DREB subfamily A-1 of ERF/AP2 transcription factor family
Chiifu	AT5G59710	VIP2	Vernalization	VIRE2 INTERACTING PROTEIN 2	Encodes a nuclear-localized NOT (negative on TATA-less) domain-containing
Turnip	AT1G53090	SPA4	Photoperiod	SPA1-RELATED 4	WD-40 and protein kinase-like domain
Turnip	AT4G27430	CIP7	Photoperiod	COP1-INTERACTING PROTEIN 7	-
Turnip	AT5G64813	LIP1	Photoperiod	LIGHT INSENSITIVE PERIOD 1	GTPase

Five lost genes are related to flowering time in Rapid cycling, covering all five categories of lowering time genes. In the other genomes just three genes are found, related only to photoperiod in turnip and vernalization in Chiifu.

### Evolutionary divergence

Chiifu, rapid cycling and turnip are estimated to have diverged 259,000 years ago, far preceding domestication (around 10,000 years ago). This may seem to imply that prior to domestication there was already considerably divergence among *B. rapa* genomes; however, domestication can accelerate selection and hence influence divergence time estimates. We do not know whether there was already variation in appearance, such as enlarged hypocotyls, leaves that form heads, multi tillering types etc. prior to domestication, or whether (more likely) there was a common wild type, and that breeders merely combined mutations and allelic variation by crossing which gave rise to diverse morphotypes. It is also unknown whether early plant breeders could breed for all different morphotypes starting from the same genetic materials, or that specific *B. rapa* materials (in geographical niches) resulted in certain morphotypes. Resequencing more *B. rapa* genotypes belonging to turnip, leafy and oil types, especially from diverse geographical regions (e.g. European and Asian turnips) may shed light on these questions.The percentage of *B.rapa* genes that have orthologs in *T. halophila* is higher than the percentage with orthologs in *A. thaliana*. The divergence date of *A. thaliana* and *B. rapa* is estimated at 17 MYA, earlier than that of *T. halophila* and *B. rapa* at 12 MYA and earlier than the whole genome triplication event dated 5–9 MYA, after speciation of *A. thalian*a/*B. rapa* and *T. halophila*/*B. rapa*[28]. In other words, *B. rapa* genes are expected to be more similar to *T. halophila* genes than to *A. thaliana* genes.

### Computational analysis

As practical considerations make it hard to obtain the sequencing depth required for *de novo* genome assembly, in this work we took a hybrid approach in which we first mapped reads to a reference genome and then created new genomes by applying all variation found. The trade-off between the number of detected variants and mapping accuracy is important. A low mapping quality threshold setting leads to many candidate genes for further experimental validation, but can also introduce false positive discoveries.

For the purposes of this study, we developed a number of scripts for variant calling, re-annotation and functional annotation that can help biologists to answer similar questions on genotype-phenotype relations. The re-annotation is a particulary time-consuming step, which may be extended by considering RNA-seq data or available gene model GFF and FASTA files.

## Conclusions

Here we present two novel reference genomes and their annotations representing the morphotypes turnip and rapid cycling to the *B. rapa* community, which provides reliable templates for studying genetic variation between these two morphotypes and the reference, Chiifu. In addition, this paper offers a complete workflow for those having limited computational resources and bioinformatics expertise studying similar biological questions. We investigated the resulting *B. rapa* pan-genome, paying specific attention to potential drivers of morphological variation. The number of genes with protein-coding changes among the three *B. rapa* genomes was lower than that among three diverse accessions of *Arabidopsis thaliana.* We found peroxidases, mainly involved in phenylpropanoid biosynthesis, enriched in the genes retained in turnip. Analysis of the gene content of the *B. rapa* pan-genome revealed that the divergence date between the three morphotypes was dated long before domestication (250,000 versus 5,000-10,000 years ago).

## Methods

### DNA material and sequencing

The genomes of a Japanese turnip doubled haploid line DH-VT117 and a rapid cycling oil-like inbred line RC-144 were resequenced. DH-VT117 is a purple red peeled round vegetable turnip derived through microspore culture from a donor plant of *B. rapa* ssp. c*ampestris* cv. Toya, CGN15201. RC-144 (Osborn FIL501) is self-compatible and has a rapid life cycle of about 24 days from sowing to flowering. Libraries with insert sizes of 300 bp, 500 bp and 2kbp were sequenced on Illumina Hiseq 2000 (Illumina Inc., San Diego CA), yielding ~100 million 71 bp paired reads for the turnip genome and ~100 million for the rapid cycling genome. Read data of the three libraries of both genotypes were independently converted to single-color binary files for fast reloading with removal of PCR duplicates, using the cortex_var assembly software tool [29]. All seven resulting binary files (including the reference genome) were then used to call variants using the Bubble Caller algorithm. Separately, the two genotypes were compared to the reference Chiifu genome using the Path Divergence Caller algorithm.

### Genome annotation

Gene level differences between turnip, rapid cycling and the reference genome (*B. rapa* ssp pekinensis, Chiifu) can only be meaningfully compared when gene models are comparable, i.e. predicted using the same method. Gene models for the reference genomecan be downloaded from the BRAD website (http://brassicadb.org/brad/), but the methods used to determine these unfortunately are not described in sufficient detail to allow reproduction of the annotation. We therefore used the MAKER genome annotation pipeline to re-annotate all three genomes [14]. Next to MAKER’s default reference gene models, all available unigenes of *B. rapa*, *Brassica napus, Brassica oleracea* were imported as closely related EST evidences and unigenes from other Brassica species as alternate evidence. Protein homology evidence was based on protein sequences of *Brassicaceae* in the NCBI RefSeq database [30]. SNAP, AUGUSTUS and GENEMARK [31–33] were used to predict genes. All other MAKER parameters were set to default values.

### Genomic variation detection

Genomic variation was detected using the cortex_var software suite with *k*-mer size set to 31, by both the Bubble Caller and Path Divergence Caller algorithms [29]. Variants found by these two algorithms are defined as *overlapping* when they either shared the same position on the chromosome or their mapped positions were close enough that one of the contigs could cover another. Such overlapping variants were merged by choosing the one yielding the longest assembled sequence. After filtering out genomic variants where the 5′ flank of the contig maps to the reference with a mapping quality score *Q* < 30, genomes of rapid cycling and turnip were reconstructed by applying all remaining variants to the reference genome. Both unmapped contigs and contigs with *Q* < 30 were annotated by MAKER, using the same settings as used for the whole genome re-annotation. The NCBI non-redundant protein database was searched for predicted gene models (BLAST, default settings) to exclude bacterial sequences. Genes on unmapped contigs with no bacterial hits were subjected to functional annotation and pathway assignment, but not included in pan genome composition because they cannot be used to detect positional orthologs.

### Common, unique and dispensable genes

Two genes found in two genomes are considered *positionally orthologous* when they are reciprocal best BLAST hits and located on the same chromosome or scaffold. We first detected such positional orthologs between *A. thaliana* and *T. halophila* on the one hand and Chiifu, turnip and rapid cycling on the other. A gene is defined as *lost* when a positional ortholog is missing in only one of the three genomes and as *retained* when it is present in only one of the three genomes (Figure 2).

Next, we detected positional orthologs between each two of the three *B. rapa* genomes. We define *common* genes as genes found in two out of three comparisons (i.e. present in all three genomes) and *unique* genes as genes not occurring in any comparison. Genes that are in neither the common gene set nor the unique gene set are called *dispensable*.

### Subgenome assignments

Genes in the Chiifu genome were assigned to the same subgenome as the one published if the new gene model mapped to the same coordinates [3]. For the other two genomes, genes were assigned to subgenomes by transferring assignment from the reciprocal best BLAST hit of Chiifu when available, or from the closest flanking genes (one gene upstream and one gene downstream) if these are in the same subgenome. Genes were not assigned to a subgenome if these rules did not apply.

### Functional annotation

To annotate genes in the *B. rapa* pan-genome, first common, dispensable and unique genes were searched in the NCBI non-redundant protein database (2012/06/07) using BLAST with default settings [34]. For dispensable and unique genes an additional InterProScan analysis was performed [35]. Gene function was predicted using the Blast2GO pipeline version B2G4Pipe version 2.5.0, integrating the BLAST and InterProScan results and KEGG pathways (based on gene EC numbers) if applicable [36]. Plant specific metabolic pathways were added as supplementary resource using the latest PlantCyc database files (release 6.0) [19].

### Candidate genes for morphological differences

Orthology between genes in *A. thaliana* and *T. halophila* on the one hand and *B. rapa* common, dispensable and unique genes on the other hand, was assessed using Inparanoid 4.1 [37]. *A. thaliana* and *T. halophila* protein sequences were downloaded from ftp://ftp.jgi-psf.org/pub/compgen/phytozome/v8.0. For each gene in *A. thaliana* and *T. halophila* the numbers of orthologous genes in Chiffu, turnip and rapid cycling were counted.

Enriched Gene Ontology terms for dispensable and unique genes without orthologs in either *A. thaliana* or *T. halophila* were determined for each genome using Fisher’s exact test [16]. Gene family information of the Brassica genes was inferred from *A. thaliana* orthologs using the latest curated gene family assignment, downloaded from TAIR (ftp://ftp.arabidopsis.org/Genes/Gene_families/).

### Inferring divergence time

In order to estimate the time of divergence of Chiifu, turnip and rapid cycling, first orthologous genes were found between these three subspecies of *B. rapa* and *A. thaliana*, *Arabidopsis lyrata*, *Oryza sativa*, *Vitis vinifera* and *Zea mays*. Orthologous relationships among the five non-*B. rapa* species were retrieved from the OMA browser [23] and only genes with a one-to-one relationship between two species (i.e. genes with only one orthologous gene in another species) were taken into account, to prevent inclusion of in-paralogs. Orthologous pairs between *A. thaliana* and *B. rapa* Chiifu were used to connect orthologous relationships between the non-*B. rapa* species and the three *B. rapa* subspecies. Multiple sequence alignments generated by EMMA (EMBOSS, [38]) using orthologous gene groups were independently analysed (i.e. with unlinked trees) under a codon-position specific estimated generalized time reversible (GTR) substitution model with lognormal relaxed clock in BEAST [39].

## Electronic supplementary material

Additional file 1: **Change rate on each chromosome and different effect types.** Excel file containing details of the change rate in turnip and rapid cycling. (XLSX 15 KB)

Additional file 2: **Functional annotation of common genes in Chiifu, turnip and rapid cycling.** Excel file containing details of the functional annotation of common genes in Chiifu, turnip and rapid cycling. (XLSX 3 MB)

Additional file 3: **The GO term enrichment analysis results on unique and dispensable genome of Chiifu, turnip and rapid cycling.** Excel file of results obtained from GO term enrichment analysis using unique and dispensable genome of Chiifu, turnip and rapid cycling. (XLSX 345 KB)

Additional file 4: **Functional annotation of dispensable, unique and unmapped genes in Chiifu, turnip and rapid cycling.** Excel file containing details of the functional annotation of dispensable, unique and unmapped genes on Chiifu, turnip and rapid cycling. The unmapped genes only apply to the turnip and rapid cycling genomes. (XLSX 1 MB)

Additional file 5: **KEGG and PlantCyc pathway assignment of dispensable and unique genes in Chiifu, turnip and rapid cycling.** Excel file containing details of KEGG and PlantCyc pathway assignments of dispensable and unique genes in Chiifu, turnip and rapid cycling. (XLSX 83 KB)

Additional file 6: **The reference genomes of turnip and rapid cycling.** Fasta file containing details of the chromosomes and scaffolds on turnip and rapid cycling. The two newly assembled genomes, their annotation files, a gene list for the three categories of pan genomes and the Blast2GO project files. (ZIP 73 MB)

Additional file 7: **The annotation files of Chiifu, turnip and rapid cycling genome.** Archived file containing three GFF files, including details of the gene features in Chiifu, turnip and rapid cycling. (ZIP 73 MB)

Additional file 8: **Common, dispensable and unique genes in Chiifu, turnip and rapid cycling.** Excel file containing details of the full complement of genes on Chiifu, turnip and rapid cycling. (ZIP 16 MB)

Additional file 9: **Blast2GO project files of dispensable and unique genes in Chiifu, turnip and rapid cycling.** Archived file containing three DAT files, each including a project file which can be imported in the Blast2GO program for further analysis. (ZIP 752 KB)

Additional file 10: **All new created scripts in the study.** Archived file containing all new created scripts used in the study. (ZIP 15 MB)

Additional file 11: **The flowering time candidate gene list in** ***A. thaliana.*** Excel file listing candidate genes related to flowering time in *A. thaliana,* categorized into flower development, gibberellin, photoperiod, vernalization and metabolic process. (XLSX 253 KB)
